# Association Between Use of Warfarin for Atrial Fibrillation and Outcomes Among Patients With End-Stage Renal Disease: A Systematic Review and Meta-analysis

**DOI:** 10.1001/jamanetworkopen.2020.2175

**Published:** 2020-04-06

**Authors:** Mandeep S. Randhawa, Rohanlal Vishwanath, Manoj P. Rai, Ling Wang, Amritpal K. Randhawa, George Abela, Gaurav Dhar

**Affiliations:** 1 Division of Cardiology, Michigan State University, Kalamazoo; 2 Sparrow Clinical Research Institute, Sparrow Healthcare, Lansing, Michigan; 3 College of Osteopathic Medicine, Michigan State University, East Lansing; 4 Department of Medicine, Michigan State University, East Lansing; 5 Division of Occupational and Environment Medicine, Michigan State University, East Lansing

## Abstract

**Importance:**

Several studies have examined the role of warfarin in preventing strokes in patients with atrial fibrillation and end-stage renal disease; however, the results remain inconclusive.

**Objective:**

To assess recently published studies to examine the outcomes of the use of warfarin among patients with atrial fibrillation and end-stage renal disease.

**Data Sources:**

A literature search was performed using the terms *warfarin and atrial fibrillation and end-stage renal disease* and *warfarin and atrial fibrillation and dialysis* in the MEDLINE, Embase, and Google Scholar databases from January 1, 2008, to February 28, 2019.

**Study Selection:**

The studies included were those with patients with end-stage renal disease and atrial fibrillation who were receiving warfarin and with hazard ratios (HRs) of at least 1 primary outcome. The studies excluded were those with a lack of information on outcomes and unreliable 95% CIs of the results.

**Data Extraction and Synthesis:**

The Meta-analysis of Observational Studies in Epidemiology (MOOSE) guidelines were followed in selecting studies. Collected data were also scrutinized for reliable 95% CIs. Finally, studies were examined for perceived biases, their limitations, and the definitions of the outcomes.

**Main Outcomes and Measures:**

The HRs and 95% CIs were calculated for the incidence of ischemic stroke, hemorrhagic stroke, major bleeding, and mortality among patients receiving anticoagulants and those not receiving anticoagulants.

**Results:**

Study selection yielded 15 studies with a total of 47 480 patients with atrial fibrillation and end-stage renal disease. Of these patients, 10 445 (22.0%) were taking warfarin. With a mean (SD) follow-up period of 2.6 (1.4) years, warfarin use was associated with no significant change for the risk of ischemic stroke (HR, 0.96; 95% CI, 0.82-1.13), with a significantly higher risk of hemorrhagic stroke (HR, 1.49; 95% CI, 1.03-1.94), with no significant difference in the risk of major bleeding (HR, 1.20; 95% CI, 0.99-1.47), and with no change in overall mortality (HR, 0.95; 95% CI, 0.83-1.09).

**Conclusions and Relevance:**

In the studies reviewed, warfarin use appears to have been associated with no change in the incidence of ischemic stroke in patients with atrial fibrillation and end-stage renal disease. However, from the studies reviewed, it does appear to be associated with a significantly higher risk of hemorrhagic stroke, with no significant difference in the risk of major bleeding, and with no change in mortality.

## Key Points

**Question:**

What is the role of warfarin in preventing ischemic strokes in patients with atrial fibrillation and end-stage renal disease?

**Findings:**

This meta-analysis found that, although the current literature is only observational, warfarin use is associated with no change in the incidence of ischemic stroke but with a significantly higher risk of hemorrhagic stroke, no significant difference in the risk of major bleeding, and no change in overall mortality.

**Meaning:**

The results suggest that warfarin use is associated with no benefit for ischemic stroke incidence or mortality and with a higher risk of hemorrhagic stroke in patients with atrial fibrillation and end-stage renal disease.

## Teaser

This meta-analysis assesses the outcomes of the use of warfarin among patients with atrial fibrillation and end-stage renal disease.

## Introduction

Atrial fibrillation (AF) is an electrophysiological anomaly causing loss of synchronized atrial contractility that puts patients at increased risk of thrombus formation and subsequent cardioembolic ischemic stroke.^1^ Anticoagulation is associated with a reduction in the incidence of ischemic stroke in patients with AF.^1^ However, the risk of ischemic stroke among patients with AF further increases with comorbidities and is associated with a higher CHA_2_DS_2_-VASc (cardiac failure or dysfunction, hypertension, age 65-74 [1 point] or ≥75 years [2 points], diabetes mellitus, and stroke, transient ischemic attack or thromboembolism [2 points]–vascular disease, and sex category [female]) score.^1,2^ With abnormal homeostasis, patients with end-stage renal disease (ESRD) are at higher risk of ischemic stroke and bleeding events.^3,4,5^ Patients with ESRD also have a higher incidence of AF compared with the general population.^6,7^

Warfarin remains a commonly used anticoagulant in the setting of ESRD. Several studies have investigated the effectiveness and outcomes of warfarin in preventing ischemic strokes in patients with ESRD and AF^8,9,10,11,12,13,14,15,16,17,18,19,20,21,22,23^; most have been either observational or retrospective, and very few have been prospective. Notably, to our knowledge, no randomized clinical trials have examined the role of anticoagulation in patients with ESRD and AF, and periodically performed meta-analyses have provided inconsistent results.^7,24,25,26,27,28,29,30,31,32^ This inconsistency has even prevailed in the guidelines from various societies. Where the American Heart Association/American College of Cardiology guideline^1^ recommends anticoagulation in patients with ESRD and AF, the European Cardiovascular Society guideline^33^ emphasizes the lack of evidence for such a recommendation, and the Kidney Disease: Improving Global Outcomes^34^ guideline recommends against the use of warfarin in such situations.

Recently, large observational and population-based studies were published on the role of warfarin for patients with ESRD and AF, which elucidated this condition.^17,18,19,20,21,23,35^ In this study, we have performed an updated meta-analysis by assessing additional information that has been made available in the last few years and using stricter inclusion criteria. Thus, to our knowledge, our study represents one of the most extensive samples of patients with AF and ESRD.

Our primary aim is to perform a systematic review of the outcomes of the observational studies and perform a meta-analysis of the available data to describe the role of warfarin for patients with ESRD who have AF. Because there have been 5 additional studies in this field in the past 2 years (with a total sample size of 24 099 patients), we believe these studies would significantly enhance the power of the data. In this meta-analysis, we have included studies that can provide specific data on 4 outcomes: ischemic stroke, hemorrhagic stroke, bleeding, and mortality.

## Methods

### Selection of Articles

Two of us (M.S.R. and M.P.R.) performed the literature search, which was further reviewed by 2 other coauthors (R.V. and A.K.R.) for any missing studies. We used the following set of terms in our search: *warfarin and atrial fibrillation and end-stage renal disease* and *warfarin and atrial fibrillation and dialysis*. We searched the MEDLINE, Embase, and Google Scholar databases from January 1, 2008, to February 28, 2019. References of review articles and studies were also manually searched for additional studies. We found 311 articles to screen for original studies that included patients with ESRD or undergoing dialysis who also had AF. The baseline characteristics of the study patients are described in eTable 1 in the Supplement. The quality analysis and bias risk assessment of the selected studies were performed using the Newcastle-Ottawa Scale^36^ (eTable 2 in the Supplement). This study followed the Preferred Reporting Items for Systematic Reviews and Meta-analyses (PRISMA) reporting guideline.^37^

### Inclusion and Exclusion Criteria

**Figure 1.**
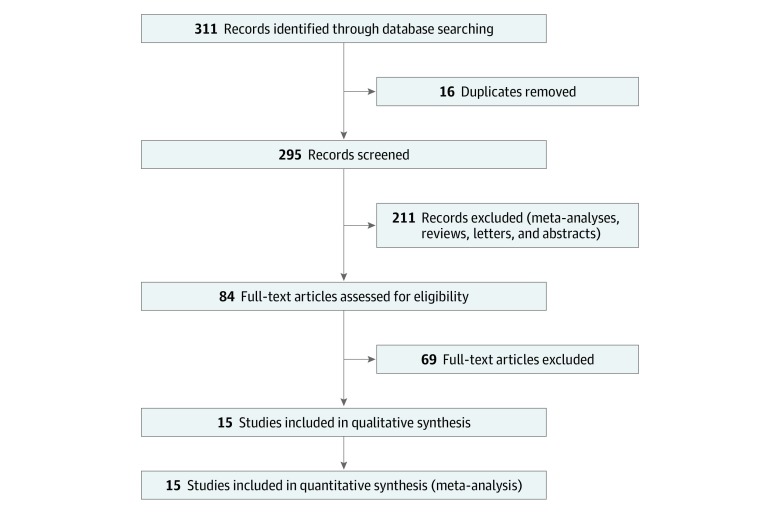
Flowchart Depicting Literature Review and Study Selection

Only the original articles that analyzed patients with ESRD or undergoing dialysis who were receivng warfarin secondary to AF were included. Patients with ESRD are defined as patients with a calculated glomerular filtration rate less than 15 mL/min and/or as patients undergoing dialysis. Patients with chronic kidney disease but not undergoing dialysis were excluded. We included studies that provided hazard ratios (HRs) of at least 1 primary outcome (ie, ischemic stroke, hemorrhagic stroke, major bleeding, or mortality). We excluded nonoriginal articles and removed duplicates. Our study excluded articles that lacked clear information on patients who were receiving warfarin. Finally, we excluded studies in which the 95% CIs of the results were not reliable based on logarithmic heterogeneity (ie, the 95% CI was too large). Fifteen unique studies were finally identified and included in this meta-analysis (Figure 1). Eleven studies were included for ischemic stroke,^8,9,11,12,13,15,16,19,20,21,22^ 7 studies were included for hemorrhagic stroke,^8,9,15,17,20,21,22^ 9 studies were included for major bleeding events,^9,10,12,13,15,16,17,19,20^ and 9 studies were included for mortality^9,13,14,15,16,19,20,22,23^ (Figure 1, Table 1,^8,9,10,11,12,13,14,15,16,17,19,20,21,22,23^ and Table 2^8,9,10,11,12,13,14,15,16,17,19,20,21,22,23^).

**Table 1.** Data on Included Studies

Source	Study type	Total study population, No.	Patients with ESRD or HD and AF	Received warfarin, No. (%)		Reported follow-up	Periods data collected
				Patients	Male patients		
Chan et al,^8^ 2009	Retrospective cohort	1671	988	508 (51.4)	294 (57.8)	Mean, 1.6 y	2003-2004
Winkelmayer et al,^9^ 2011	Retrospective cohort	2313	2313	249 (10.7)	106 (42.6)	Mean, 1.76 y	1994-2006
Carrero et al,^10^ 2014	Prospective, observational	24 317	478	66 (13.8)	41 (62.1)	Not reported	2003-2010
Chen et al,^11^ 2014	Nationwide cohort analysis	4899	4899	294 (6.0)	122 (41.5)	Mean, 1503 d	1995-2008
Genovesi et al,^14^ 2015	Prospective cohort	290	290	134 (46.2)	86 (64.2)	Median, 4 y	2010
Shah et al,^12^ 2014	Retrospective cohort	205 836	1626	756 (46.5)	461 (61.0)	Not reported	1998-2007
Wakasugi et al,^13^ 2014	Prospective cohort	60	60	28 (46.6)	16 (57.0)	Total, 110 person-years	2008-2011
Shen et al,^15^ 2015	Retrospective cohort	12 284	12 284	1838 (14.9)	913 (49.7)	Total, 16 617 person-years	2007-2011
Garg et al,^16^ 2016	Retrospective cohort	302	302	119 (39.4)	66 (55.4)	Mean, 2.1 y	2009-2012
Wang et al,^17^ 2016	Retrospective cohort	774	141	59 (41.8)	36 (61.0)	Mean, 4.4 y	2000-2008
Kai et al,^20^ 2017	Retrospective cohort	4286	4286	989 (23.1)	625 (63.2)	Mean, 2.1 y	2006-2015
Lee et al,^22^ 2017	Retrospective cohort	6719	2356	589 (25.0)	249 (42.3)	Mean, 2 y	2000-2010
Tan et al,^19^ 2019	Retrospective cohort	5765	5765	1651 (28.6)	710 (43.0)	Not reported	2007-2011
Yoon et al,^21^ 2017	Retrospective nonrandomized	9974	9974	2921 (29.2)	1750 (59.9)	Mean, 15.9 mo	2009-2013
Voskamp et al,^23^ 2018	Prospective cohort	1718	1718	244 (14.2)	146 (59.8)	Total, 5 y	Not reported
Total	NA	281 208	47 480	10 445 (22.0)	5621 (53.8)	NA	NA

Abbreviations: AF, atrial fibrillation; ESRD, end-stage renal disease; HD, hemodialysis; NA, not applicable.

**Table 2.** Studies With Ischemic Stroke, Hemorrhagic Stroke, Major Bleeding, and Mortality Outcomes

Source	Sample size, No.		Ischemic stroke			Hemorrhagic stroke			Major bleeding			Mortality		
	Warfarin	Control	No. (%)		HR (95% CI)	No. (%)		HR (95% CI)	No. (%)		HR (95% CI)	No. (%)		HR (95% CI)
			Warfarin	Control		Warfarin	Control		Warfarin	Control		Warfarin	Control	
Chan et al,^8^ 2009	508	480	158 (31.2)	63 (13.1)	1.81 (1.12-2.92)	33 (6.5)	14 (2.9)	2.22 (1.01-4.91)	NA	NA	NA	NA	NA	NA
Winkelmayer et al,^9^ 2011	249	2064	29 (12.2)	135 (14.2)	0.92 (0.61-1.37)	11 (4.6)	46 (2.2)	2.38 (1.15-4.96)	48 (20.3)	215 (22.7)	0.96 (0.70-1.31)	181 (76.3)	750 (79.0)	1.06 (0.90-1.24)
Carrero et al,^10^ 2014	66	412	NA	NA	NA	NA	NA	NA	4 (6.1)	34 (8.3)	0.52 (0.16-1.65)	NA	NA	NA
Chen et al,^11^ 2014	294	2983	16 (5.6)	119 (4.0)	1.02 (0.67-1.53)	NA	NA	NA	NA	NA	NA	NA	NA	NA
Genovesi et al,^14^ 2015	134	156	NA	NA	NA	NA	NA	NA	NA	NA	NA	75 (56.0)	95 (61.0)	0.91 (0.56-1.48)
Shah et al,^12^ 2014	756	870	52 (6.9)	55 (6.3)	1.14 (0.78-1.67)	NA	NA	NA	149 (19.7)	125 (14.4)	1.41 (1.09-1.81)	NA	NA	NA
Wakasugi et al,^13^ 2014	28	32	8 (28.5)	5 (15.6)	1.94 (0.63-5.93)	NA	NA	NA	3 (10.7)	4 (12.5)	0.85 (0.19-3.64)	9 (32.1)	9 (28.1)	1.00 (0.40-2.52)
Shen et al,^15^ 2015	1834	10446	62 (3.4)	501 (4.8)	0.68 (0.47-0.99)	29 (1.6)	188 (1.8)	0.82 (0.37-1.81)	153 (8.3)	888 (8.5)	1.00 (0.69-1.44)	831 (45.2)	4596 (44.0)	1.01 (0.92-1.11)
Garg et al,^16^ 2016	119	183	13 (10.9)	21 (11.4)	0.93 (0.49-1.82)	NA	NA	NA	26 (22.0)	26 (14.2)	1.53 (0.94-2.51)	97 (81.5)	145 (79.2)	1.03 (0.91-1.15)
Wang et al,^17^ 2016	59	82	NA	NA	NA	4 (6.8)	0	11.11 (1.15-107.2)	11 (18.6)	5 (6.1)	3.26 (1.13-9.4)	NA	NA	NA
Kai et al,^20^ 2017	989	3297	67 (6.7)	304 (9.2)	0.68 (0.52-0.90)	2 (2.0)	45 (1.4)	1.2 (0.6-2.2)	126 (13.0)	368 (11.0)	0.97 (0.77-1.2)	495 (50.0)	1813 (55.0)	0.76 (0.69-0.84)
Lee et al,^22^ 2017	589	1767	48 (8.1)	51 (2.9)	0.92 (0.57-1.48)	6 (1.0)	35 (2.0)	0.84 (0.32-2.19)	NA	NA	NA	340 (57.7)	1050 (59.4)	1.04 (0.88-1.23)
Tan et al,^19^ 2019	1651	4114	93 (5.6)	646 (15.7)	0.88 (0.70-1.11)	NA	NA	NA	406 (24.6)	1555 (37.8)	1.48 (1.32-1.66)	475 (28.8)	3349 (81.4)	0.72 (0.65-0.80)
Yoon et al,^21^ 2017	2921	7053	222 (7.6)	458 (6.5)	1.09 (0.93-1.28)	88 (3.0)	141 (2.0)	1.44 (1.10-1.88)	NA	NA	NA	NA	NA	NA
Voskamp et al,^23^ 2018	244	1474	NA	NA	NA	NA	NA	NA	NA	NA	NA	141 (57.7)	538 (36.5)	1.20 (1.00-1.50)

Abbreviations: HR, hazard ratio; NA, not applicable.

### Data Extraction and Management

Two of us (M.S.R. and M.P.R.) independently searched the databases using the search techniques described. Furthermore, we analyzed the published meta-analyses to look for missed studies. The selected studies were reviewed for type of study, year published, total population, mean age, percentage of patients taking warfarin, percentage of patients taking antiplatelet agents, percentage of male participants, and HRs of the outcomes. Extracted data were collected using standardized collection forms, and tables were created for the outcomes. Disagreement regarding inclusion or exclusion criteria were resolved with consensus. Collected data were further scrutinized for reliable 95% CIs by the statistician (L.W.). We used Meta-analysis of Observational Studies in Epidemiology (MOOSE) reporting guideline for assessing data quality.^54^ Finally, we also looked for the perceived biases mentioned in the studies, their limitations, and the definitions of the objectives and outcomes (eTable 3 in the Supplement).

### Statistical Analysis

All statistical analyses were performed using Stata, version 15.0, statistical software (StataCorp LP). Statistical analyses for adjusted risks of outcomes were performed. With survival data, log HR and its variance were calculated based on the adjusted risk of outcomes. The overall HRs were pooled by a random-effects model. Heterogeneity was assessed using the Cochran *Q* test and the inconsistency or *I*^2^ statistic; for the *Q* statistic, *P* < .10 was considered statistically significant for heterogeneity, whereas for the *I*^2^ statistic, a value greater than 50% was considered statistically significant for heterogeneity. Publication bias was examined by use of a funnel plot of each study’s effect size against the precision (1/SE). Publication bias arises when trials with statistically significant results are more likely to be published and cited, and are preferentially published in English-language journals and those indexed in MEDLINE.^38^ The publication bias was assessed by use of the Egger test at *P* < .10.^39^ Finally, the sensitivity analyses were performed using the leave-1-out approach.

## Results

Fifteen unique studies with a total of 47 480 patients with AF and ESRD were analyzed and included in this meta-analysis. Of these patients, 10 445 (22.0%) received warfarin (Table 1).^8,9,10,11,12,13,14,15,16,17,19,20,21,22,23^ Among the patients receiving anticoagulants, 5621 (53.8%) were male. The mean (SD) follow-up period was 2.6 (1.4) years.

### Ischemic Stroke

**Figure 2.**
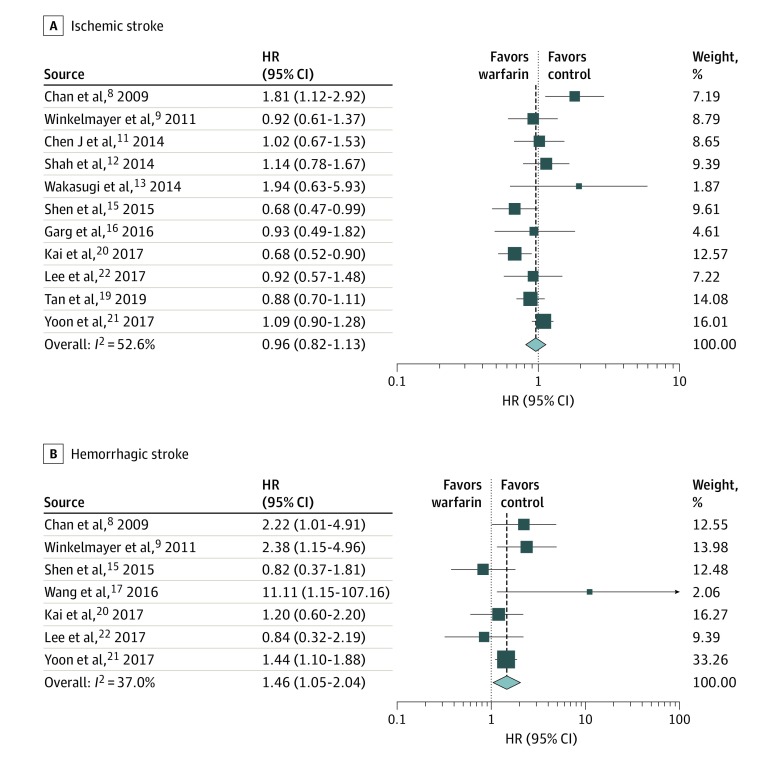
Forest Plots Showing Hazard Ratios (HRs) of Ischemic Stroke and Hemorrhagic Stroke

The 11 studies of ischemic stroke included 43 231 patients, of whom 9942 (23.0%) were receiving warfarin.^8,9,11,12,13,15,16,19,20,21,22^ Of the 9942 patients receiving warfarin, 768 (7.7%) had an ischemic stroke; of the 33 289 patients who were not receiving warfarin, 2358 (7.1%) had an ischemic stroke. The overall HR for ischemic stroke was 0.96 (95% CI, 0.82-1.13), indicating no benefit of using warfarin for patients with ESRD and AF in preventing ischemic strokes (Table 2,^8,9,10,11,12,13,14,15,16,17,19,20,21,22,23^ Figure 2).

### Hemorrhagic Stroke

The sample size for hemorrhagic stroke was a total of 32 342 patients from 7 studies, with 7153 patients (22.1%) receiving warfarin.^8,9,15,17,20,21,22^ The hemorrhagic stroke rate for patients who took warfarin was 2.4% (n = 173). In contrast, the rate of hemorrhagic stroke for patients not taking warfarin was 1.9% (469 of 25 189), with an overall HR for hemorrhagic stroke of 1.46 (95% CI, 1.05-2.04) (Table 2,^8,9,10,11,12,13,14,15,16,17,19,20,21,22,23^ Figure 2). These values indicate an association between warfarin use and a higher risk of hemorrhagic stroke.

### Major Bleeding

**Figure 3.**
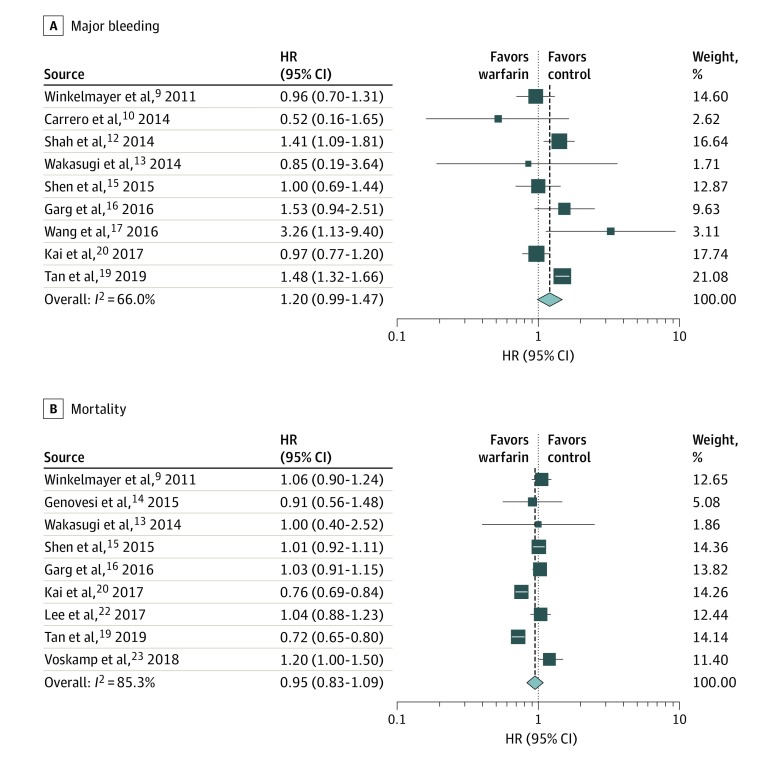
Forest Plots Showing Hazard Ratios (HRs) of Major Bleeding and Mortality

The sample size for major bleeding events was a total of 27 251 patients from 9 studies, with 5751 patients (21.1%) taking warfarin.^9,10,12,13,15,16,17,19,20^ The rate of major bleeding events was 16.1% (n = 926) for patients who took warfarin compared with 15.0% (3220 of 21 500) for patients not taking warfarin. The overall HR for major bleeding was 1.20 (95% CI, 0.99-1.47) (Table 2,^8,9,10,11,12,13,14,15,16,17,19,20,21,22,23^ Figure 3). These values indicate no association of anticoagulation with major bleeding events.

### Mortality

Of the 9 studies on mortality, the sample size was 29 623, with 6090 patients (20.6%) who received warfarin.^9,13,14,15,16,19,20,22,23^ The mortality rate was 43.4% (2644) for the patients who took warfarin and 52.5% (12 345 of 23 533) for the patients who did not take warfarin, with an overall HR of 0.95 (95% CI, 0.83-1.09) (Table 2,^8,9,10,11,12,13,14,15,16,17,19,20,21,22,23^ Figure 3). This suggest that overall mortality does not seem to be associated with anticoagulation for these patients.

### Sensitivity Analysis

Results for the leave-1-out sensitivity analyses are shown in eTable 4 in the Supplement. The overall HR remained consistent based on sensitivity analysis for most variables, except for the overall HR for hemorrhagic stroke, which is less precise. The HR for hemorrhagic shock varied from 1.35 (95% CI, 0.95-1.92) after removing the study by Winkelmayer et al^9^ to an HR of 1.55 (95% CI, 1.08-2.21) after removing the study by Lee et al.^22^

### Publication Bias

There was no evidence of publication bias by the Begg test. The *P* values were *P* = .84 for the Begg tests for ischemic stroke, .81 for mortality, .19 for major bleeding, and .88 for hemorrhagic stroke.

### Measure of Heterogeneity Using Cochran *Q* and *I*^2^ Statistics

The studies analyzed for the primary outcome of mortality were mostly heterogeneous, with an *I*^2^ of 85.3% indicating a high inconsistency in data. It was followed by studies of major bleeding, with an *I*^2^ of 66.0%, and studies of ischemic stroke, with an *I*^2^ of 52.6% indicating a moderate level of inconsistency in the data. The least inconsistency was for studies of hemorrhagic stroke, with an *I*^2^ of 37.0%. The results were similar using the Cochran *Q* test: *P* < .01 for studies analyzing mortality, *P* = .003 for studies of major bleeding, *P* = .02 for studies of ischemic stroke, and *P* = .15 for studies of hemorrhagic stroke (eTable 4 in the Supplement).

## Discussion

We performed a meta-analysis of one of the largest sample sizes, to our knowledge, of patients with AF and ESRD. Using definitions that defined outcomes, quality of data, and prevailing biases, we included only studies that met our criteria (Table 1).^8,9,10,11,12,13,14,15,16,17,19,20,21,22,23^ Warfarin use was associated with with no significant change in the risk of ischemic stroke, with a significantly higher risk of hemorrhagic stroke, with no significant difference in risk of major bleeding, and with no change in overall mortality.

### Comparison With Other Meta-analyses

In the contemporary period, several meta-analyses have been performed in different parts of the world.^7,24,25,26,27,28,29,30,31,32^ With the latest studies, our data substantiate the results of these analyses, suggesting that there is no association between warfarin use for patients with ESRD and AF and protection against ischemic strokes but rather an association between warfarin use and a higher risk of hemorrhagic stroke.^15,24,25,26,28^ Lee et al,^26^ Lui et al,^24^ and Nochaiwong et al^27^ showed that there was no association of warfarin use with ischemic stroke and mortality but that there was an association of warfarin use with an increased risk of hemorrhagic stroke and major bleeding. However, previous studies have also shown conflicting results, and few studies have actually demonstrated an increased risk of stroke with the use of warfarin by patients with AF and ESRD,^8,40^ while other studies demonstrated neither an increased risk of ischemic stroke nor any benefit of using warfarin in reducing the risk of ischemic stroke.^10^

### Anticoagulation in Patients With AF and ESRD

Various studies and meta-analyses have helped us to understand the role of renal dysfunction in patients with AF. The meta-analysis by Dahal et al^25^ shows that warfarin use is associated with a reduction in the incidence of stroke and mortality, with no change in the incidence of bleeding in patients with chronic kidney disease. However, the study by Dahal et al^25^ also shows that, once patients developed ESRD, warfarin use was associated not with a decreased risk of stroke and mortality but with an increased risk of major bleeding. Furthermore, the study by Shih et al,^41^ using competing risk analysis, demonstrated that the risk of stroke was only modestly higher among patients with new-onset AF undergoing hemodialysis than among those without AF, and the risk became nonsignificant when accounting for the competing risk of in-hospital death. Also, this analysis showed that the value of the CHA_2_DS_2_-VASc score for ischemic stroke was also diminished in analyses, thus indicating a minimal association of the overall stroke rate with onset of AF in patients with ESRD in whom baseline stroke risk is already very high.

Similar findings have been shown in some epidemiologic studies as well. The study by Genovesi et al^42^ shows that the risk of stroke among patients receiving hemodialysis, depending on the presence of AF or sinus rhythm, proved to be inconclusive. This finding is partly also due to reported data that the patients died even before they developed the stroke itself. Moreover, the major cause of stroke among patients with ESRD is of vascular origin, thus removing any beneficial effect of anticoagulation itself for many patients in this population. The causes of death for patients with ESRD include cardiovascular disease (39%), infectious diseases (12%), stroke (10.3%), and neoplastic diseases (10%).^43^ However, patients with AF and ESRD have an even higher mortality rate than patients with ESRD without AF.^44,45,46^

### Antiplatelet Therapy in ESRD

Even though 12 of the 15 studies included in our meta-analysis mention that both controls and patients taking warfarin were receiving antiplatelet therapy at some point, only 2 studies actually measured outcomes in patients who were receiving antiplatelet therapy.^8,11^ Chan et al^8^ assessed the risk of stroke in patients with ESRD and AF. Compared with nonuse, warfarin use was associated with a significantly increased risk for new stroke (HR, 1.93; 95% CI, 1.29-2.90), and there was no association of warfarin use with overall mortality. A higher incidence of strokes was mainly due to hemorrhagic strokes, especially among patients whose international normalized ratio was not monitored in the first 90 days. Futhermore, the use of the antiplatelet agent aspirin or clopidogrel alone was not associated with a lower risk of stroke compared with nonusers (aspirin: HR, 0.86; 95% CI, 0.56-1.32; clopidogrel: HR, 0.66; 95% CI, 0.30-1.46). Chen et al^11^ reported a similar finding. Patients with ESRD who were treated with antiplatelet agents did not have an overall lower risk of ischemic stroke compared with control groups (HR, 0.83; 95% CI, 0.57-1.21). Both studies also validate the data reported in our analysis, that warfarin use was associated with no benefite in reducing the incidence of ischemic stoke in patients with ESRD.

### Randomized Clinical Trials and Anticoagulation in ESRD

The only controlled studies that are performed in the setting of ESRD and warfarin use are those on preventing vascular access failure due to thrombosis.^47,48,49^ These studies did not show any significant benefit in the prevention of vascular access failure compared with the risk of bleeding. Moreover, one study showed that acetylsalicylic acid was as effective in the prevention of vascular access failure due to thrombosis as warfarin, but without the severe complications.^49^ Therefore, based on expert opinion, the routine use of warfarin in the prevention of vascular access failure due to thrombosis in patients with ESRD is not recommended.^50^

### Left Atrial Appendage Occlusion Devices in Patients With ESRD and AF

Finally, left atrial appendage occlusion (LAAO) devices could be a promising alternative for patients with ESRD and AF for stroke prevention. PROTECT AF (Percutaneous Left Atrial Appendage Closure for Stroke Prophylaxis in Patients With Atrial Fibrillation), the first randomized clinical trial using the WATCHMAN device, showed an overall reduction in both mortality and stroke among patients with AF.^51^ The US Food and Drug Administration approved the WATCHMAN device in 2015 for patients with AF who cannot tolerate long-term oral anticoagulation. Comparison between LAAO devices and oral anticoagulation in patients with chronic kidney disease was investigated by Kefer et al^52^ using an Amplatzer cardiac plug (ACP). The team of Kefer et al^52^ demonstrated a similar procedural safety for LAAO devices in patients with chronic kidney disease and normal renal function and a significant reduction in risk of stroke and risk of major bleeding events.

Similarly, Genovesi et al^53^ have shown the safety of the LAAO device in patients with ESRD. Overall, the use of LAAO devices has been shown to be effective in reducing strokes. Their use in patients with reduced renal function is safe and effective, which provides a newer direction for further investigations of LAAO devices in patients with ESRD and AF.

In the end, this meta-analysis provides evidence that the use of warfarin for patients with AF and ERSD was associated with no change in the incidence of ischemic stoke. Anticoagulants other than warfarin including non–vitamin K antagonist oral anticoagulants, antithrombotics or antiplatelets with or without warfarin, and LAAO devices should be investigated for use among patients with AF and ESRD.

### Limitations

Our study has some limitations. In the absence of randomized clinical trials, our study’s retrospective and observational nature. First, there is significant clinical heterogeneity, which weakens the results of our meta-analysis. The definition of the end points is variable in the different studies, as outlined in eTable 3 in the Supplement. The difference between 2 groups of patients, those treated with warfarin and those not treated with warfarin, is not clearly defined in the original data. The number of patients in each group receiving antithrombotic therapy is variable and not clearly defined in study cohorts except for 2 studies by Chan et al^8^ and Chen et al.^11^ In addition, several patients were not taking warfarin, likely owing to underlying increased risk for bleeding or adverse events such as previous bleeding. Our analysis found variable statistical heterogeneity, with maximum heterogeneity identified among studies included for the primary outcome of mortality (*I*^2^ = 85.3%), followed by major bleeding (*I*^2^ = 66.0%) and ischemic stroke (*I*^2^ = 52.6%). Thus, the use of the random-effects model was appropriate for this analysis. Second, there was a lack of data to risk stratify the cohorts based on baseline characteristics and comorbidities. Third, the studies included in this meta-analysis were required to have at least 1 of the primary outcomes established. Therefore, the studies were unequally distributed in each of the 4 outcomes of our analysis. A few included studies did not directly include patients with ESRD or hemodialysis; rather, the data obtained were from a subset of the population included in these studies.

## Conclusions

The evidence available regarding the use of warfarin for the prevention of ischemic stroke in patients with AF in the setting of ESRD is observational and conflicting, and data from randomized clinical trials not are available to date. Available data show that warfarin use is not associated with any benefit in the prevention of ischemic stroke. Instead, it is associated with a significant increase in the risk of hemorrhagic stroke, no significant difference in the risk of major bleeding, and no association with overall mortality.

## Notes

Supplement. **eTable 1.** Baseline Characteristics of the Study Population **eTable 2.** Newcastle Ottawa Scale Assessment of the Studies **eTable 3.** Definitions of Major Bleeding, Ischemic Strokes, and Hemorrhagic Strokes as Mentioned in the Selected Studies **eTable 4.** Sensitivity Analysis With “Leave Out” Method and Measure of Heterogeneity Using Cochran’s Q and I^2^ Results
