# Supplementary material for: Association Between Use of Warfarin for Atrial Fibrillation and Outcomes Among Patients With End-Stage Renal Disease: A Systematic Review and Meta-analysis
Source: JAMA Netw Open. 2020 Apr 6;3(4):e202175. doi: 10.1001/jamanetworkopen.2020.2175 (PMC7136833; doi:10.1001/jamanetworkopen.2020.2175)
Supplement: Supplement. — eTable 1. Baseline Characteristics of the Study Population eTable 2. Newcastle Ottawa Scale Assessment of the Studies eTable 3. Definitions of Major Bleeding, Ischemic Strokes, and Hemorrhagic Strokes as Mentioned in the Selected Studies eTable 4. Sensitivity Analysis With “Leave Out” Method and Measure of Heterogeneity Using Cochran’s Q and I2 Results [file jamanetwopen-3-e202175-s001.pdf]

Supplementary Online Content

Randhawa MS, Vishwanath R, Rai MP, et al. Association between use of warfarin for atrial fibrillation and outcomes among patients with end-stage renal disease: a systematic review and meta-analysis. *JAMA Netw Open.* 2020;3(4):e202175.
doi:10.1001/jamanetworkopen.2020.2175

eTable 1. Baseline Characteristics of the Study Population
eTable 2. Newcastle Ottawa Scale Assessment of the Studies
eTable 3. Definitions of Major Bleeding, Ischemic Strokes, and Hemorrhagic Strokes as Mentioned in the Selected Studies
eTable 4. Sensitivity Analysis With "Leave Out" Method and Measure of Heterogeneity Using Cochran's Q and I^2 Results

This supplementary material has been provided by the authors to give readers additional information about their work.

© 2020 Randhawa MS et al. *JAMA Network Open.*

eTable 1. Baseline Characteristics of the Study Population

Authors	Mean age		Male (%)		Hypertension (%)		Diabetes Mellitus (%)		Coronary artery disease (%)		History of stroke (%) (I, H, NS)**	
	Warfarin	Control	Warfarin	Control	Warfarin	Control	Warfarin	Control	Warfarin	Control	Warfarin	Control
**Chan et al[8]**	72.6	71.3	57.8	54.4	79.7	79.8	40.6	34.4	58.3	52.9	14.4(NS)	11.9(NS)
**Winkelmayer et al[9]**	68.6	70.1	42.6	42.5	82.7	80.7	60.2	59.1	46.2	53.0	29.9(NS)	23.6(NS)
**Carrero et al[10]**	-	-	-	-	-	-	-	-	-	-	-	-
**Chen J et al[11]**	-	-	41.5	46.3	81.0	83.1	45.9	47.6	61.9	54.9	-	-
**Genovesi et al[14]**	-	-	64.2	56.4	76.1	85.3	29.1	33.3	45.5	50.6	15.7(I), 11.9(H)	14.1(I), 26.3(H)
**Shah et al[12]**	75.3	75.1	61.0	61.0	77.0	75.0	44.0	39.0	62.0	59.0	6.0(NS)	5.0(NS)
**Wakasugi et al[13]**	67.8	68.4	57	72	43	28	18	28	-	-	14(I)	26(I)
**Shen et al[15]**	61.2	62.1	49.7	48.7	97.2	98.6	69.1	70.8	37.0	42.6	22.0(NS)	26.8(NS)
**Garg et al[16]**	75	78	55.4	51.3	84.8	88.0	58.8	55.1	77.3	80.9	20.1(I)	23.0(I)
**Wang et al[17]**	59.8	62.1	61	62.2	98.3	89.0	66.1	54.9	71.2	72.0	27.1(NS)	15.9(NS)
**Kai et al[20]**	69.2	67.3	63.2	58.9	99.2	99.0	79.2	77.9	55.1	49.5	24.0(I), 1.3(H)	25.2(I), 2.5(H)
**Lee et al[22]**	69.3	70.3	42.3	40.6	-	-	-	-	-	-	6.8(NS)	5.9(NS)
**Tan et al[19]**	74.4	74.7	43	43.2	98.4	98.6	70.7	71.6	65.9	66.4	21.0(NS)	21.6(NS)
**Yoon et al[21]**	67.8	66.1	59.9	57.5	89.4	79.2	43.1	35.9	-	-	-	-
**Voskamp et al[23]**	67.6	61.3	59.8	61.6	88.1	89.1	17.6	15.8	59.0	31.7	-	-

* *Where 'I' is ischemic, 'H' is hemorrhagic and 'NS' is not specified

© 2020 Randhawa MS et al. *JAMA Network Open.*

eTable 2. Newcastle Ottawa Scale Assessment of the Studies

Study	***Selection***	***Compatibility***	***Outcomes***
**Chan et al[8]**	****	-	*
**Winkelmayer et al[9]**	****	*	**
**Carrero et al[10]**	**	-	-
**Chen J et al[11]**	****	*	**
**Genovesi et al[14]**	****	**	***
**Shah et al[12]**	***	*	*
**Wakasugi et al[13]**	****	**	***
**Shen et al[15]**	****	*	***
**Garg et al[16]**	****	*	***
**Wang et al[17]**	****	**	***
**Kai et al[20]**	****	*	***
**Lee et al[22]**	****	*	***
**Tan et al[19]**	****	*	-
**Yoon et al[21]**	****	*	***
**Voskamp et al[23]**	****	**	***

© 2020 Randhawa MS et al. *JAMA Network Open.*

eTable 3. Definitions of Major Bleeding, Ischemic Strokes, and Hemorrhagic Strokes as Mentioned in the Selected Studies

Definition of Major Bleeding		
Defined as	Number of Studies	Studies
Hospitalization/transfusion	5	Carrero et al[10], Wakasugi et al[13], Shen et al[15], Garg et al[16], Wang et al[17].
Internal bleeding	2	Winkelmayer et al[9], Shah et al[12].
Not defined	1	Genovesi et al[14].
Definition of Ischemic Stroke		
Defined as	Number of Studies	Studies
ICD codes	5	Winkelmayer et al[9], Chen J et al[11], Lee et al[22], Tan et al[19], Yoon et al[21].
Clinical evidence	2	Wakasugi et al[13], Garg et al[16].
Others	3	Chan et al[8], Shah et al[12], Kai et al[20]
Not defined	1	Shen et al[15]
Definition of Hemorrhagic Stroke		
Defined as	Number of Studies	Studies
ICD codes	3	Winkelmayer et al[9], Lee et al[22], Yoon et. Al[21]
Radiographic evidence	1	Wang et al[17]
Others	2	Chan et al[8], Shah et al[12]
Not defined	1	Shen et al[15]

© 2020 Randhawa MS et al. *JAMA Network Open.*

eTable 4. Sensitivity Analysis With “Leave Out” Method and Measure of Heterogeneity Using Cochran’s Q and I^2 Results

	Sensitivity Analysis				***Measures of heterogeneity***			
	Remove Winkelmayer’s et al[10] & Wakasugi et al[11]	*Remove Shen et al[15], Shah et al[12] & Lee et al[22]*	*Remove Wakasugi et a1[11], Winkelmayer et al[10], Chan et al[9], & Shen et al[15]*	*Remove Tan et al[19], Wakasugi et al[11], Shah et al[12] & Yoon et al[21]*	*Cochran’s Q (p-value)*	*I^2 (%)*	*Modified H^2*	*Tau^2*
Ischemic Stroke	0.95 (0.81-1.12)	0.997 (0.85 - 1.17)	0.97 (0.81 – 1.57)	0.948 (0.796 – 1.13)	21.09 (0.02)	52.60%	1.109	0.034
Hemorrhagic Stroke	1.35 (0.95-1.92)	1.55 (1.08 -2.21)	1.38 (0.95 – 1.99)	1.49 (0.917 – 2.45)	9.53 (0.146)	37.00%	0.588	0.0686
Major Bleeding	1.25 (1.01-1.55)	1.16 (0.914 -1.488)	1.24 (0.99 – 1.54)	1.21 (0.986 – 1.49)	23.54 (0.003)	66.00%	1.943	0.0465
Mortality	0.94 (0.81-1.08)	0.94 (0.9 -1.94)	0.95 (0.83 – 1.09)	0.99 (0.877 – 1.12)	54.56 (<0.01)	85.30%	5.82	0.0301

© 2020 Randhawa MS et al. *JAMA Network Open.*
